# Limited english proficiency and concordance of CKD awareness among primary care providers and patients

**DOI:** 10.1186/s12882-020-02155-3

**Published:** 2020-12-10

**Authors:** Alexis F. Velazquez, Alexandra Velasquez, Delphine S. Tuot

**Affiliations:** 1 School of Medicine, University of California, San Francisco, USA; 2 Division of Nephrology, University of California, San Francisco, USA; 3 Center for Vulnerable Populations, Priscilla Chan and Mark Zuckerberg San Francisco General Hospital and Trauma Center, University of California, 1001 Potrero Ave, Bldg. 100, Room 342, San Francisco, CA 94110 USA

**Keywords:** CKD, Awareness, Kidney disease, Limited English proficiency

## Abstract

**Background:**

Patient awareness of CKD and primary care provider (PCP) recognition of CKD are lower than for other chronic conditions. Understanding how patients may become aware of CKD is critical to their participation in healthy behaviors to slow CKD progression. We examined factors associated with the concordance of CKD awareness among patients and providers and hypothesized that concordance of CKD awareness would be influenced by social and demographic factors that impact communication, such as limited English proficiency (LEP) and health literacy.

**Methods:**

Between July 2011 to July 2014, patients with CKD from three primary care clinics in a public healthcare delivery system were surveyed with questions regarding their health, including awareness of their CKD status. Chart review was performed to identify PCP recognition of CKD, defined as CKD listed anywhere in the problem list within nine months before patient enrollment into the study. We used logistic regression to determine the association between provider recognition and patient awareness of kidney disease among those patients with CKD, adjusting for patient demographics, co-morbidities, and provider training.

**Results:**

The study population (*n* = 152) had a mean age of 57.4 (SD 13), was 48.7% male and was racially/ethnically and linguistically diverse: 89.5% self-identified as Black, Hispanic, or Asian and 32.2% had LEP. Most patients had hypertension (89.5%) and diabetes (77.6%); mean eGFR was 66.1 ml/min/1.73m^2^ (SD 32.8). Positive concordance of CKD awareness was 42% (*n* = 64). Odds of positive concordance with their providers were much higher among patients with LEP compared to English speaking patients (adjusted odds ratio = 11.07, 95%CI 1.60–76.39).

**Conclusions:**

Concordance of CKD awareness among PCPs and their patients with CKD in one public delivery system was higher among patients with LEP. While speculative, this may be due to greater caution in provider communication about CKD with LEP patients.

**Supplementary Information:**

The online version contains supplementary material available at 10.1186/s12882-020-02155-3.

## Background

As the nationwide prevalence of diabetes and hypertension increases, so has the prevalence of Chronic Kidney Disease (CKD) [1]. Early kidney disease is asymptomatic and requires awareness of risk factors in order to be tested and subsequently diagnosed with blood or urine tests. This is likely one reason why CKD awareness among patients with mild CKD is very low, with national estimates of awareness among individuals with Stages 1–2 CKD (defined by an estimated glomerular filtration rate [eGFR] > 60 ml/min/1.73m^2^ and abnormal levels albuminuria) of 3.2% [2]. Even awareness of CKD among patients with moderate-severe CKD (stages 3–4, defined by an eGFR 15–59 ml/min/1.73m^2^) is low, with national estimates of 10.5% [3]. Contributing to this low awareness is suboptimal recognition of patients’ kidney disease among providers, as measured by documentation of CKD in the electronic health record. In one large managed care cohort of over 10,000 patients, primary care provider documentation of CKD with administrative codes among patients with lab-diagnosed CKD stages 1–4 was only 14.4% [4]. Similarly, one study of 466 primary care practices geographically distributed in the U.S. identified that PCP documentation of CKD among patients with lab-diagnosed CKD and type 2 was 12.1% [5].

CKD is associated with a high risk of cardiovascular disease, and if left untreated, can progress to End Stage Renal Disease (ESRD), which is in turn associated with early mortality [6]. CKD awareness among patients is hypothesized to influence adherence to therapies and participation in self-management behaviors known to help delay CKD progression. Thus, understanding how patients become aware of CKD is important to better direct awareness efforts. Patient awareness of CKD probably depends on provider awareness of CKD, as they can diagnose the disease and discuss treatment during a clinical encounter. Data from the Awareness, Detection and Drug Therapy in type 2 Diabetes and Chronic Kidney Disease (ADD-CKD) study support this hypothesis, as analyses found that provider documentation of CKD was associated with patient awareness of their CKD status [5].

With this study, we sought to examine factors associated with the concordance of CKD awareness among patients and providers. We hypothesized that the concordance of CKD awareness would be influenced by social and demographic factors that impact communication, such as limited English proficiency and health literacy.

## Methods

### Study design, setting, and participants

This is a cross-sectional study that examines the concordance of provider and patient awareness of CKD status in one public healthcare delivery system, leveraging data collected for a study that examined different questions to ascertain CKD awareness among patients. Detailed methods from that study have been published previously [7]. Briefly, patients were recruited from three primary care clinics in the integrated public health care delivery system for a large city in California. After receiving consent by primary care providers to approach their patients, patients were recruited in a clinic by a language-concordant member of the research team. Eligible patients were required to be over the age of 18, speak English, Spanish, or Cantonese, have been seen at least once in their primary care clinic within the 9 months prior to the study, and have CKD or one of three other chronic medical conditions: hypertension, diabetes or hyperlipidemia. Patients who could not read, had significant vision impairment, or could not actively participate in a discussion with health care providers about chronic medical conditions (e.g. very hard of hearing, dementia, active psychosis) were excluded from this study.

A total of 211 patients consented to participate in the original study. Three patients were excluded because they did not include sufficient information on their questionnaire, fourteen patients were excluded because they did not have a visit with their PCP within the 9 months before the survey was administered, and two additional patients were excluded because they had ESRD. Of the remaining 192 participants, 152 were identified to have CKD and were included in this study population. This study was approved by the Institutional Review Board at the University of California, San Francisco (#10–00771).

### Data collection and definitions

After patients provided written consent to participate, they were administered a language appropriate, in-person health questionnaire with a language concordant study team member that included questions about their demographics and assessment of their overall health, as well as about co-morbidities that contribute to kidney disease such as diabetes and hypertension. Questions regarding demographics asked patients to identify their age, sex, education level, insurance, and race/ethnicity. Health literacy was determined with a validated three-item instrument [8] that measured reading literacy and included the following questions with Likert-style response scales: (1) “How often do you have someone help you read hospital materials?”; (2) “How confident are you filling out medical forms by yourself?”; and (3) “How often do you have problems learning about your medical conditions because of difficulty understanding written information?” An individual was considered to have limited health literacy if he or she had a score > 6 of a maximal score of 12 [9]. The survey also asked a series of questions to assess patients’ understanding of their CKD status and risk factors (Supplemental Figure 1). There is no universally accepted definition for limited English Proficiency, thus our health questionnaire used the question, “What language do you primarily speak at home?” for patients to self-identify as having limited English Proficiency.

After we identified patients and administered the questionnaire, we performed a systematic chart review to identify PCP recognition of CKD, PCP demographic information, and patient medical information. All clinical notes from primary care patient visits within 9 months of the patient’s completion of the questionnaire were reviewed for provider awareness of CKD. We assumed that patients with at least one chronic disease would have seen their PCP at least once in a 9-month period. CKD awareness was defined as CKD or use of any keywords about CKD listed in the assessment/plan section of documented clinical encounter [3]. Keywords included: kidney disease, CKD, CRF, renal disease, proteinuria, nephropathy, declining GFR, renal lesion, followed by renal, albuminuria (macro or micro) or renal insufficiency. Provider awareness of other health conditions, including hypertension, diabetes, and hyperlipidemia, were defined in a similar fashion. Hypertension keywords included “HTN”, “hypertension”, “elevated or high BP”. Diabetes keywords included “diabetes”, “DM”, “DM2”, “DM1”. Hyperlipidemia keywords included “high/elevated lipids”, “HL”, “hyperlipidemia”, “dyslipidemia”.

We then abstracted patient data, including the most recent laboratory test results within the two years prior to patient enrollment as well as medication prescriptions to determine the presence or absence of chronic health conditions. CKD was defined by two values of MDRD eGFR < 60 ml/min/1.73m^2^ separated by a minimum of 3 months or the presence of persistent proteinuria, defined by two values of albuminuria > 30 mg/g or > 1+ dipstick proteinuria over a minimum 3-month period of time. Diabetes was defined by a glycosylated hemoglobin (A1C) > 6.5% or prescription of diabetes medications that were most commonly used in the health care delivery system (Insulin, Metformin, Glyburide or Glipizide). Hyperlipidemia was defined by an LDL cholesterol level > 130 mg/dL or prescription of a statin medication. Hypertension was defined by the last clinic blood pressure > 140/90 mmHg or prescription of Angiotensin Coenzyme inhibitors, Angiotensin II Receptor Blockers, Beta-Blocker, Calcium Channel Blockers, or Loop Diuretics.

### Statistical analysis

The primary outcome was positive concordance of CKD awareness between patient and provider, defined by both the provider and the patient being aware of the patient’s CKD status. We first identified variables associated with the two individual components of concordance of CKD awareness – patient awareness and provider awareness – using chi-squared univariate analysis. Then, we used multivariable logistic regression to determine which of those factors were associated with the positive concordance of awareness among individuals with CKD. Model 1 adjusted for patient demographics, including age, sex, limited English proficiency (LEP), educational attainment, type of health insurance (Medicaid, Medicare, None) and self-reported language concordance with PCP. Model 2 further adjusted for co-morbid conditions associated with CKD awareness in the univariate analyses (systolic blood pressure and eGFR). In Model 3 (our final regression model) we further adjusted for provider type (Attending Physician, Resident trainee or Nurse Practitioner /Physician Assistant).

To better understand the results of the concordance analysis, we used similar multivariable logistic regression models to analyze factors associated with the individual components of concordance, namely patient awareness of CKD and provider awareness of CKD. For each outcome, model 1 adjusted for patient demographics, model 2 additionally adjusted for co-morbidities and model 3 further adjusted for provider type.

## Results

### Participant demographics

The overall study population of 152 patients had a mean age of 57.4 years and was evenly balanced with respect to gender (48.7% were male). It represented the racial/ethnic diversity of the patient population receiving care in the public delivery system, with the majority of patients identifying as Hispanic (37.5%), Black (44.1%) or Asian (7.9%) (Table 1). Over 30% of the patient population spoke a primary language other than English, of which about 90% was Spanish. Over three-fourths (76%) of study participants reported usual professional interpreter use or language concordance with their provider, including only 27% of patients who were LEP. Approximately 30% of patients had limited health literacy. Above 60% of the patient population had a high school degree or less. The majority of patients were insured under Medicare or Medicaid (73.7%). Most patients had hypertension (89.5%) and diabetes (77.6%) and nearly two-thirds had CKD stages 3–4 (63.8%). Providers whose clinical notes were included in the study were 64% attending physicians, 21% resident physicians, and 15% nurse practitioners or physician assistants.

**Table 1:** Characteristics of the study population

Characteristics	All (n = 152)	English proficient (***n*** = 103)	Limited English Proficient (***n*** = 49)	***p***-value
Mean Age, years (SD)	57.4 (13.6)	57.9 (11.9)	56.5 (16.8)	0.56
Male, % (N)	48.7 (74)	54.4 (56)	36.7 (18)	0.04
Race/Ethnicity, % (N)				< 0.01
Non-Hispanic White	9.2 (14)	13.6 (14)	0	
Hispanic	37.5 (57)	9.7 (10)	95.9 (47)	
Black	44.1 (67)	65.0 (67)	0	
Asian	7.9 (12)	9.7 (10)	4.1 (2)	
Other	1.3 (2)	1.9 (2)	0	
Language, % (N)				< 0.01
English	67.8 (103)	103 (100)	0	
Spanish	30.9 (47)	0	95.9 (47)	
Cantonese	0.6 (1)	0	2.0 (1)	
Other	0.6 (1)	0	2.0 (1)	
Provider-Patient Language Concordance, % (N)	76.3 (116)	100 (103)	26.5 (13)	0.04
Adequate Health Literacy, % (N)	69.3 (104)	71.6 (73)	64.6 (31)	0.39
Educational Attainment, % (N)				< 0.01
Primary School	15.1 (23)	2.9 (3)	40.8 (20)	
High School/Vocational School	46.7 (71)	49.5 (51)	40.8 (20)	
College	38.2 (58)	47.6 (49)	18.4 (9)	
Health Insurance, % (N)				< 0.01
None	20.9 (30)	10.4 (10)	42.6 (20)	
Medicaid	55.9 (80)	65.6 (63)	36.2 (17)	
Medicare	23.1 (33)	23.9 (23)	20.4 (10)	
Hypertension, % (N)	90.1 (136)	93.1 (95)	83.7 (41)	0.07
Mean Systolic BP, (SD)	134.7 (22.2)	137.5 (21.9)	128.8 (21.6)	0.03
Mean Diastolic BP, (SD)	78.9 (12.4)	81.2 (13.2)	74.0 (8.6)	< 0.01
Mean eGFR (SD)	66.1 (32.8)	62.6 (29.1)	73.3 (38.8)	0.06
CKD stage, % (N)				0.24
Stage 1–2	36.2 (55)	33.0 (34)	42.9 (21)	
Stage 3–4	63.8 (97)	66.9 (69)	57.1 (28)	
Proteinuria, % (N)	77.5 (107)	78.9 (71)	75.0 (36)	0.6
Diabetes, % (N)	77.6 (118)	79.61 (82)	73.47 (36)	0.4
Mean Cholesterol (LDL) Mg/dL, (SD)	83.0 (46.0)	82.0 (43.6)	85.1 (50.9)	0.7

n = 152 for all rows except SBP (*n* = 148), DBP (n = 148), Insurance (n-143), Cholesterol (n = 130)

### Concordance of CKD awareness

Among the 152 patients with CKD, 57% (*n* = 86) demonstrated concordance of awareness with their primary care provider, of which 64 patients and providers were both aware of the patient’s CKD status (positive concordance) and 22 where both provider and patient were unaware (negative concordance). Approximately one-half of patients with CKD whose providers did not document CKD in the clinical documentation (*n* = 25/47) demonstrated awareness of their CKD status (Table 2). In multivariate analysis, the results of which are depicted in Table 3, limited English proficiency independently conferred higher odds of having a positive concordance of CKD awareness with one's PCP compared to English proficiency (adjusted odds ratio (aOR): 11.07; 95% CI: 1.60–76.39). CKD severity was also associated with higher odds of positive concordance of CKD awareness (aOR for each unit decrease of eGFR = 1.02; 95% CI: 1.01–1.03). Women had a significantly lower odds of positive CKD concordance with their providers compared to men (aOR: 0.42; 95% CI: 0.17–0.99) as did patients with Medicare insurance compared to those with no insurance (aOR 0.19; 95% CI: 0.04–0.96).

**Table 2:**
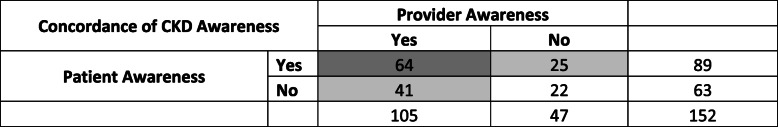
Concordance of chronic kidney disease awareness among providers and patients with chronic kidney disease by laboratory measurements. Positive concordance is shaded in dark gray (*n* = 64); discordance is shaded in light gray (*n* = 66)

**Table 3:** Odds of CKD Concordance among PCPs and their patients with CKD with complete data (*n* = 130)

Characteristics	Model 1	Model 2	Model 3
**Age (per year increase)**	0.99 (0.95–1.02)	0.98 (0.94–1.01)	0.98 (0.94–1.01)
**Female sex**	**0.43 (0.19–0.97)**	**0.42 (0.18–0.99)**	**0.42 (0.17–0.99)**
**Limited English Proficiency**	**6.92 (1.24–38.57)**	**11.07 (1.63–74.93)**	**11.07 (1.60–76.39)**
**Educational Attainment**			
Primary School	**Ref**	**Ref**	**Ref**
High School/Technical School	1.22 (0.29–5.19)	1.64 (0.35–7.76)	1.61 (0.32–8.22)
College	1.84 (0.42–8.12)	2.59 (0.51–13.25)	2.55 (0.48–13.5)
**Health Insurance**			
None	ref	ref	ref
Medicaid	1.06 (0.33–3.38)	1.04 (0.30–3.57)	1.05 (0.29–3.72)
Medicare	0.24 (0.05–1.02)	**0.19 (0.04–0.95)**	**0.20 (0.04–0.96)**
**Provider-Patient Language Concordance**	1.59 (0.31–8.15)	2.08 (0.37–11.77)	2.07 (0.37–11.77)
**Systolic Blood Pressure**		0.99 (0.98–1.02)	0.99 (0.98–1.02)
**eGFR (per ml/min/1.73 m**^**2**^ **increase)**		**0.98 (0.97–0.99)**	**0.98 (0.97–0.99)**
**Provider Type**			
Attending physician			Ref
Resident trainee			0.97 (0.34–2.77)
Nurse practitioner/Physician Assistant			1.02 (0.32–3.19)

**Model 1:** patient demographics (age, sex, LEP status, Insurance type, educational attainment, language concordance with provider)

**Model 2:** Model 1 + systolic blood pressure, estimated glomerular filtration rate (eGFR)

**Model 3:** Model 2 + provider type

### Provider and patient awareness of CKD

Over two-thirds of patients (69%; *n* = 105/152) had at least one note that documented PCP awareness of their patients’ CKD status. In univariate analyses, patient age, eGFR, limited English Proficiency and provider type were associated with higher odds of provider documentation of their patients’ CKD status. All of these associations were attenuated in the fully adjusted model, such that only female sex (aOR: 0.41; 0.17–0.94) and lower eGFR (aOR per ml/min/1.73m^2^ increase: 0.99; 0.97–1.00) were highly suggestive of being associated with provider documentation of CKD status (Table 4).

**Table 4:** Odds and 95% confidence intervals of factors associated with patient awareness and provider awareness of chronic kidney disease among patients with CKD

Characteristics	Odds (95% CI) of Patient Awareness of CKD	Odds (95% CI) of Provider Awareness of CKD
Age (per year increase)	**0.97 (0.93–1.00)**	0.97 (0.93–1.01)
Female sex	0.75 (0.35–1.61)	**0.41 (0.17–0.94)**
Limited English Proficiency	2.55 (0.51–12.67)	7.47 (0.92–60.52)
Education		
Primary School	Ref	Ref
High School/Technical School	0.91 (0.23–3.55)	3.62 (0.71–18.46)
College	0.98 (0.24–4.01)	4.92 (0.93–26.04)
Insurance		
None	Ref	Ref
Medicaid	0.92 (0.29–2.89)	0.59 (0.14–2.38)
Medicare	0.38 (0.99–1.44)	0.59 (0.12–2.99)
Provider-Patient Language Concordance	2.06 (0.46–9.17)	1.78 (0.25–12.39)
Systolic Blood Pressure	0.99 (0.98–1.02)	1.01 (0.99–1.03)
eGFR (per ml/min/1.73 m2 increase)	**0.99 (0.97–0.99)**	**0.99 (0.97–1.00)**
Provider Type		
Attending physician	Ref	Ref
Resident trainee	1.83 (0.69–4.86)	1.29 (0.45–3.72)
Nurse practitioner/Physician Assistant	0.81 (0.29–2.18)	1.45 (0.42–4.96)

Multivariable models include all of the variables listed

Nearly 59% of patients (*n* = 89/152) were aware of their CKD. Patient awareness of CKD status seemed to differ by provider type in the univariate model, with patients treated by resident physicians having 2-fold higher odds (aOR: 2.14; 0.92–4.96) of CKD awareness than patients treated by an attending physician. In the same model, patients insured with Medicare had lower odds of CKD awareness than those with other insurance (aOR: 0.29; 0.10–0.84). However, both associations were attenuated when adjusting for patient demographic factors and co-morbid conditions. In the final multivariable model, only younger patient age (aOR for each additional year: 0.97; 0.93–1.00) and eGFR (aOR = 0.99; 0.97–0.99) were independently associated with higher odds of CKD awareness among patients (Table 4).

## Discussion

The main finding in this study was that primary care patients with CKD in one public healthcare delivery system had higher odds of being positively concordantly aware of their CKD status with their PCP if they were limited English proficient (compared to English proficient). Since the concordance of CKD awareness depends on communication between the provider and patient about kidney health, it is not surprising that a patient characteristic that directly informs communication (LEP status) was independently associated with concordance. However, the direction of the results was surprising, as prior studies have suggested that limited English proficiency (as well as less formal education, racial minority, and low health literacy) negatively impacts health outcomes and quality of care [10]. A large systematic review of 33, mostly cross-sectional, studies of the impact of provider and patient language non-concordance on health outcomes suggests that language non-concordance often presents as a barrier to optimal patient health [11]. In this review, studies of primary care outcomes showed better glycemic control, reduction in LDL, and lower blood pressure with language concordant care; studies of hospital care outcomes demonstrated increased patient satisfaction with care, fewer ED visits upon discharge, and better outpatient transition.

Although we did not find that a patient’s LEP status was associated with greater CKD awareness by individual patients, results suggest that when a patient has limited English proficiency, there is a change in the dynamic of interaction that occurs between the patient and provider that may improve concordant awareness. There could be several reasons for this finding. First, it’s probable that during primary care visits when CKD was identified in the clinical documentation, providers used greater care and time when explaining a diagnosis of CKD to their LEP patients (vs. English speaking patients). For example, they could have used more robust communication techniques to educate patients and their families, such as using less complicated vocabulary, providing a summary at the end of the visit, allowing time to answer patient questions and using teach-back methods [12]. In our study population, limited English proficiency was highly suggestive of being monolingual Spanish speaking and was strongly associated with Hispanic ethnicity. It is well known that Hispanics have a greater risk for end stage kidney disease compared with Whites [13]. This may have prompted providers to spend additional time discussing kidney disease with their patients of Hispanic origin, many of whom also happened to have limited English proficiency.

Additionally, the healthcare delivery system has long invested in robust interpretation services to facilitate conversations with limited English proficient patients. A recent study demonstrated that LEP status did not negatively impact receipt of guideline-concordant CKD care in healthcare settings equipped to address potential health inequities associated with language, largely due to interpretation services [14]. In our study, around 75% patients identified language concordant care delivery or professional interpreter use during their primary care clinic, visits, supporting this idea. It is thus possible that professional interpreters help bridge cultural differences in communication styles among providers and patients when there is no language concordance, despite data from prior studies suggesting that patients are often less satisfied with clinical interactions when professional interpreters are required [15]. Provider communication about CKD is generally suboptimal in the United States, perhaps due to language used by providers to describe kidney disease [7]. It’s possible that interpreters may have enhanced understanding of kidney disease among patients by using more readily understandable words. Non-provider related communication about CKD by other members of the primary care team warrants further study.

Our evaluation of factors associated with individual patient awareness of CKD confirms data previously documented in the prior literature; younger age and more severe kidney disease are both associated with higher patient awareness [3]. We also found that patients had non-statistically higher odds of being aware of their CKD if their providers were Resident physicians (trainees) compared to attending physicians. It is unclear why patients seen with Resident physicians may have demonstrated more awareness of kidney disease, though we suspect that Resident physicians likely spent more time with patients compared with attending physicians, which could have translated into greater odds of patient awareness.

CKD severity was also associated with greater provider documentation of CKD, a factor that has also been associated with provider awareness of CKD in prior studies [3]. Interestingly, we found that trainees and Nurse Practitioners/Physician Assistants had non-statistically significant higher odds of documenting their patients’ CKD status compared to Attending physicians. Although this was not the primary focus of this study, it does suggest an important role of mid-level providers in improving the health outcomes of patients with CKD and warrants additional investigation [16].

As this study is a secondary analysis of data collected previously for a separate study, there were several limitations as to the types of variables used in our analysis. These limitations include the inability to account for unmeasured confounders related to communication such as preferred provider language, provider documentation of use of professional interpreter services and the length of time spent discussing kidney disease. In addition, using documentation as a proxy for provider awareness comes with the assumption that providers document accurately and completely after patient visits, which may not always be the case. Both these reasons may explain why 25 of 89 patients were aware of their CKD despite PCP unawareness per our study protocol. Due to the limitations of the electronic health record system used at the time of this study, we were unable to determine if a patient had been seen by a primary care or a specialist outside the network. Had a patient seen an outside PCP or emergency provider or a specialist or had only recently established care with a new PCP, it would be possible for them to be aware of their CKD status while their PCP was unaware. Additionally, we used PCP notes within the last nine months to determine PCP awareness of CKD; it’s possible that CKD was documented in notes associated with prior visits, which would not be captured by our study protocol. With regards to assessing health literacy, it’s important to note that we used a tool designed to assess reading literacy, and not numeracy, which provides a partial assessment of health literacy. When discussing CKD with a patient, a patient’s ability to understand and interpret the clinical relevance of numbers, such as eGFR or creatinine, is likely important. Although the questionnaire used in this study was piloted with patients before collecting data, the questions regarding a patient’s relationship with their provider had not been validated previously. We also did not have information about patient family history of kidney disease, whether an educated family member or care-taker was present during visits, whether a family member served as an ad-hoc interpreter, or the length of time a patient had kidney disease, all of which are factors that could contribute to concordance of CKD awareness. As with all cross-sectional studies, we cannot infer causation, only association.

## Conclusions

In summary, CKD is prevalent, complex and comes with a substantial risk for further disease and mortality. Awareness of CKD among patients remains low and there is still an incomplete understanding of how individuals become aware of their CKD diagnosis. This study suggested that provider awareness of CKD, defined by documentation in recent clinical notes, is not always associated with patient awareness of CKD but that limited English proficiency among patients plays a role in positive concordance of CKD awareness. Although inconclusive, it is clear that language is an important factor in a patient understanding their CKD diagnosis. Future research should investigate how to use these findings in interventions to enhance provider-patient discussions about CKD, thus enhancing patient awareness of kidney disease.

## Supplementary Information

**Additional file 1.**

## Data Availability

Data analyzed for this study are available upon request from corresponding author.

## Abbreviations

CKD: Chronic Kidney Disease
SFHN: San Francisco Health Network
PCP: Primary Care Provider
ESRD: End Stage Renal Disease
