# Supplementary material for: Limited english proficiency and concordance of CKD awareness among primary care providers and patients
Source: BMC Nephrol. 2020 Dec 10;21:538. doi: 10.1186/s12882-020-02155-3 (PMC7727194; doi:10.1186/s12882-020-02155-3)
Supplement: Supplementary file 1 — Additional file 1. [file 12882_2020_2155_MOESM1_ESM.pdf]

Patient awareness of chronic disease

Thank you for agreeing to participate in this survey about health-care provider - patient communication. Your answers will help us improve our communication strategies between physicians and nurses and you.

Please note that you may or may not have any of the illnesses or diseases mentioned below. Please answer the questions as accurately as possible.

Section I

Tracking Number ______________________________

1. What is the medical record number listed on your hospital card? ______________________________

2. How would you describe your race?
- ☐ Caucasian or White
- ☐ African American or Black
- ☐ Chinese
- ☐ Filipino
- ☐ Asian Indian
- ☐ Native Hawaiin or other Pacilfic islander
- ☐ Other Asian
- ☐ Other __________

3. How would you describe your ethnicity?
- ☐ Not of Hispanic or Latino origin
- ☐ Mexican, Mexican American, or Chicano
- ☐ Other Hispanic or Latino origin

4. What is your country of birth? ______________________________

5. What is the highest grade or year of school you have completed?
- ☐ Primary school (finished around age 12)
- ☐ Secondary school (finished around age 18)
- ☐ Some college
- ☐ Finished college
- ☐ Attended and/or finished graduate school

6. What language do you primarily speak at home?
- ☐ English
- ☐ Spanish
- ☐ Cantonese
- ☐ Mandarin
- ☐ Tagalog
- ☐ Russian
- ☐ Other ____________

7. In which language do you communicate with your doctor?
- ☐ English
- ☐ Spanish
- ☐ Cantonese
- ☐ Mandarin
- ☐ Tagalog
- ☐ Russian
- ☐ Other ____________

8. How well do you speak English?
- ☐ Not at all
- ☐ Not well
- ☐ Well
- ☐ Very well

9. Does a professional interpreter usually help you and your primary care provider communicate during your visit?
- ☐ Yes
- ☐ No

Section III.

The following set of questions refers to your general knowledge of disease. They are not about you or your own health.

20. Have you ever heard about kidney disease?
☐ Yes
☐ No

21. How or from whom did you hear about kidney disease, in general?

A. ... from your primary care provider? (physician or nurse)

☐ Yes ☐ No

B ... from a family member?

☐ Yes ☐ No

C ... from a friend?

☐ Yes ☐ No

D ... from a community or church group?

☐ Yes ☐ No

E ... during a community kidney disease screening event?

☐ Yes ☐ No

F ... Other? ___________________________

☐ Yes ☐ No

22. Have you ever heard about diabetes?
☐ Yes
☐ No

23. How or from whom did you find out about diabetes in general?

A ... from your primary care provider? (doctor or nurse)

☐ Yes ☐ No

B ... from a family member?

☐ Yes ☐ No

C ... from a friend?

☐ Yes ☐ No

D ... from a community or church group?

☐ Yes ☐ No

E ... during a community diabetes screening event?

☐ Yes ☐ No

F ... Other? __________________

☐ Yes ☐ No

Section III.

Now, let's move onto questions about your own health.

32. In general, would you say your health is:
- ☐ Excellent
- ☐ Very good
- ☐ Good
- ☐ Fair
- ☐ Poor

33. Have you ever been told by a doctor or other health care provider .... that you have diabetes or problems with high blood sugar?
- ☐ Yes
- ☐ No
- ☐ Unsure

34. Have you ever been told by a doctor or health care provider: ... that you have weak or failing kidneys (excluding stones, bladder infections, or incontinence?_
- ☐ Yes
- ☐ No
- ☐ Unsure

35. Have you ever been told by a doctor or other health care provider ... that you have heart disease?
- ☐ Yes
- ☐ No
- ☐ Unsure

36. Have you ever been told by a doctor or other health care provider ... that you have kidney disease?
- ☐ Yes
- ☐ No
- ☐ Unsure

37. Have you ever been told by a doctor or other health care provider ... that you have high blood pressure?
- ☐ Yes
- ☐ No
- ☐ Unsure

38. Have you ever been told by a doctor or health care provider that you have protein in the urine?
- ☐ Yes
- ☐ No
- ☐ Unsure

39. Have you ever been told by a doctor or other health care provider ... that you have had a heart attack of myocardial infarction?
- ☐ Yes
- ☐ No
- ☐ Unsure

40. Have you ever been told by a doctor or other health care provider.... that you have a kidney problem?
- ☐ Yes
- ☐ No
- ☐ Unsure

41. Have you ever been told by a doctor or other health care provider .... that you have high cholesterol?
- ☐ Yes
- ☐ No
- ☐ Unsure

42. Have you ever been told by a doctor or other health care provider.... that your kidneys are damaged?
- ☐ Yes
- ☐ No
- ☐ Unsure

43. If you have ever been told that you have a kidney problem, have you been told why? If yes, please tell me whether the following conditions are harming your kidneys.
- ☐ Yes
- ☐ No

A. ... High blood pressure?

☐ Yes ☐ No

B. ... Diabetes?

☐ Yes ☐ No

C. ... Medications?

☐ Yes ☐ No

D. ... Auto-immune disease such as lupus?

☐ Yes ☐ No

E. ... have cysts in your kidneys?

☐ Yes ☐ No

F. ... Other reason? __________

☐ Yes ☐ No

44. When you describe your kidney problem to your family or friends, what do you say?

____

45. Has anyone in your immediate family (parents, brothers, sisters, children) ever been told he or she has kidney failure? This would be someone who is on or had been on dialysis or had a kidney transplant?

☐ Yes
☐ No
☐ Unsure

Over the last 2 weeks, how often have you been bothered by any of the following problems?

46. .... little interest or pleasure in doing things?

☐ Not at all
☐ Several days
☐ More than half the days
☐ Nearly everyday

47. ... feeling down, depressed, or hopeless

☐ Not at all
☐ Several days
☐ More than half the days
☐ Nearly everyday
